# Systematic Review of Screening and Surveillance Programs to Protect Workers from Nanomaterials

**DOI:** 10.1371/journal.pone.0166071

**Published:** 2016-11-09

**Authors:** Mary Gulumian, Jos Verbeek, Charlene Andraos, Natasha Sanabria, Pieter de Jager

**Affiliations:** 1 Department of Toxicology and Biochemistry, National Institute for Occupational Health, National Health Laboratory Service, Johannesburg, South Africa; 2 Finnish Institute of Occupational Health, Helsinki, Finland; 3 Department of Epidemiology and Surveillance, National Institute for Occupational Health, National Health Laboratory Service, Johannesburg, South Africa; 4 School of Public Health, Faculty of Health Science, University of the Witwatersrand, Johannesburg, South Africa; Brandeis University, UNITED STATES

## Abstract

**Background:**

Screening and surveillance approaches for workers exposed to nanomaterials could aid in early detection of health effects, provide data for epidemiological studies and inform action to decrease exposure. The aim of this review is to identify such screening and surveillance approaches, in order to extract available data regarding (i) the studies that have successfully been implemented in present day, (ii) identification of the most common and/or toxic nano-related health hazards for workers and (iii) possible exposure surveillance markers. This review contributes to the current understanding of the risk associated with nanomaterials by determining the knowledge gap and making recommendations based on current findings.

**Methods:**

A systematic review was conducted. PubMed and Embase were searched to identify articles reporting on any surveillance-related study that described both exposure to nanomaterials and the health indicators that were measured. Four reviewers worked in pairs to independently assess the eligibility of studies and risk of bias before extraction of data. Studies were categorised according to the type of study and the medical surveillance performed, which included the type of nanomaterial, any exposure details provided, as well as health indicators and biomarkers tested.

**Results:**

Initially 92 studies were identified, from which 84 full texts were assessed for eligibility. Seven studies met all the inclusion criteria, i.e. those performed in Taiwan, Korea, Czech Republic and the US. Of these, six compared health indicators between exposed and unexposed workers and one study described a surveillance program. All studies were at a high risk of bias. Workers were exposed to a mix of nanomaterials in three studies, carbon-based nanomaterials in two studies, nano-silver in one study and nano-titanium oxide in the other study. Two studies did not find a difference in biomarkers between exposed and unexposed workers. In addition, differences in early effects on pulmonary function or neurobehavioral tests were not observed. One study found an increased prevalence of allergic dermatitis and “sneezing” in the exposed group.

**Conclusions:**

This review of recently published data on surveillance studies proves that there is a gap in the current knowledge, where most of the surveillance-related studies reported do not follow a set format that provides the required information on ENM characterisation, the type of exposure and the measured indicators/biomarkers. Hence, there is very low quality evidence that screening and surveillance might detect adverse health effects associated with workplace exposure. This systematic review is relevant because it proves that, although surveillance programs have been initiated and preliminary results are being published, the current studies are actually not answering the important questions or solving the overall problem regarding what the potential health hazards are among workers either handling or potentially exposed to ENMs. The recommendations, thus proposed, are based on an obvious need for (i) exposure registries, where longitudinal follow-up studies should inform surveillance, (ii) known exposure measurements or summary indices for ENMs as a reference (iii) validation of candidate biomarkers and (iv) studies that compare the effects of these surveillance approaches to usual care, e.g. those commonly followed for bulk-size hazardous materials.

## Introduction

The novel physicochemical properties of engineered nanomaterials (ENMs) allow new applications in biomedical, optical and electronic fields, which have lead to increased synthesis and production and ultimately increased risk of exposure. The exposure risks are associated with handling ENMs during production/manufacturing or use of ENM-enabled products, e.g. in packaging, cosmetics and sunscreens, sporting goods, tires, stain-resistant clothing, food additives, solar cells or catalysis, chemical polishing agents from semi-conductor wafers, coatings to dissipate and minimize static electricity in fuel lines and hard disk handling trays, flame-retardant fillers for plastics, field emitter sources in flat panel displays, biological cellular imaging in living cells and tissues [1,2].

Although ENMs are increasingly produced and utilized in a wide range of products, the health and environmental effects remains uncertain [3]. The study of nanotoxicology has been proposed to address the adverse health effects caused by ENMs [4]. Following the precautionary principle, a component of which includes taking preventive action in the face of uncertainty [5], workplace screening and surveillance for workers exposed to ENMs could potentially help early detection of health effects, serve as a source of data to evaluate epidemiological links between exposure and health outcomes, and, inform action to prevent disease due to exposure to ENMs.

The most common ENMs encountered in the work place and/or the environment include titanium dioxide (TiO_2_) [6], cerium dioxide [7], silicon dioxide (SiO_2_) [8], zinc oxide (ZnO) [9], silver (Ag) [10] and carbon nanotubes (CNTs) [11]. The most common exposure and entry routes for ENMs include inhalation, ingestion and dermal absorption, where putative target organs include the lung, liver, heart and brain [12]. Since human skin and lungs (even the gastro-intestinal tract) are constantly in contact with the environment, exposure time to any possible toxic agent is increased. While the skin is generally an effective barrier to foreign substances, the lungs and gastro-intestinal tract are more vulnerable. In addition, *in vivo* administration or implants are other possible routes of exposure for intentionally administered ENMs [13]. The most common concern with ENMs involves their ability to translocate from the site of deposition to other organs, such as brain, blood, liver, spleen and kidneys. ENMs that are not excreted and/or degraded may then bio-accumulate in different target organs. Therefore, it is important to consider their short-term toxicity, as well as, their long-term pathogenicity.

Occupational health surveillance has been defined as “…the ongoing systematic collection, analysis and dissemination of exposure and health data on groups of workers for the purpose of early detection of disease and injury” [14]. Occupational health surveillance consist of environmental (exposure) surveillance and medical surveillance, a component of which is medical screening [15]. Medical surveillance involves the continuous systematic collection, analysis and interpretation of specific health events (clinical monitoring) and/or changes in/of biological functioning (biological monitoring). Medical screening is a complementary activity to surveillance aiming to detect early signs of work-related illness by administering tests to apparently healthy persons. Screening activities typically have a clinical focus when compared to surveillance, but medical screening data that is collected utilizing a standardized approached, aggregated and analysed longitudinally lends itself to be evaluated as a part of a surveillance program [15]. The purpose of a surveillance programme is: i) to serve as an early warning system; ii) to monitor the effectiveness of health interventions over time and; iii) to conduct research and inform public health policy.

Some studies recommend medical screening of workers exposed to certain well-studied ENMs. For example, in the case of CNTs and carbon nanofibres (CNF), the National Institute for Occupational Safety and Health (NIOSH) states that enough toxicological evidence has been collected in recent years to allow recommendations for medical screening in the work place [3]. These recommendations focus on respiratory health care and include spirometry tests and baseline chest X-rays. Currently, there are gaps in the information provided by surveillance programs that have recently been implemented, where preliminary findings have been reported. Although the studies are considered to be an advancement in medical screening of workplace exposures, a number of challenges emerge when considering the design of screening and surveillance programs required for workers who are exposed to the broader class of ENMs in general. These include the heterogeneity of ENMs; the lack of information available on the type and quantity of ENM exposure; a lack in understanding of various exposure routes, absorption, biotransformation and excretion of ENMs, the uncertainty of translating *in vitro* and *in vivo* animal studies to possible human adverse health effects, a poor understanding of the health outcomes associated with exposure to various ENMs, as well as, a lack in validated tests to be utilized in biological monitoring for asymptomatic exposed workers [5]. Further to this, it is not clear whether or not the benefits of a targeted surveillance program for ENMs would outweigh the costs. Thus, there are a number of questions concerning the affordability, acceptability and relevance of screening and surveillance programs for ENMs in the workplace.

## Methods

A systematic review was conducted to identify worker health surveillance and screening approaches that could be implemented for workers exposed to specific ENMs or groups of ENMs. The complete study protocol is included in S1 Text. The search, constructed on concepts for: (1) health surveillance, (2) nanomaterials and (3) work, was combined into search strings to locate studies on health surveillance or screening and exposure to ENMs at work using PubMed and Embase (See S2 Text for the full search strategy). A snowballing approach was subsequently undertaken by which the bibliographies of retrieved articles were assessed for additional reports on ENM-specific surveillance programs.

All articles, abstracts or letters published after 1 January 2000 up to June 2015 without any language limitations, were eligible for inclusion. Where appropriate, updates including up to June 2016 were integrated for those studies that had already been selected as suitable in the initial search. All study designs were assessed in this review, these included descriptive studies, as well as, comparative studies that compared the effects of a health surveillance or screening program to either no or an alternative program including cross sectional, case-control, cohort and randomized control trials.

Relevant studies included any systematic collection and/or analysis of ENM-specific occupational health and safety information, which aims to monitor health status of workers potentially exposed to ENM in the workplace. Therefore, studies were excluded if they did not assess or describe the systematic collection and/or analysis of ENM-specific occupational health and safety information, which aimed to identify exposure to ENMs or to monitor or screen the health status of workers potentially exposed to ENMs in the workplace. Secondly, studies that did not report the internal (biological monitoring) and/or clinical signs and symptoms (clinical monitoring) of workers involved with the synthesis and/or application and/or handling of any nanomaterials for commercial or research purposes, were also excluded. Finally, studies that did not provide sufficient information to identify the ENMs workers are exposed to or that did not provide information on the methods used to take systematic measurements for the surveillance programme (i.e. biological and clinical measures) were excluded from the analysis.

The primary outcomes of this review were i) a description of surveillance and screening programs; ii) any reported health outcomes; the secondary outcomes included i) the cost and ii) coverage of the surveillance or screening program. Four reviewers worked in pairs to independently assess the full text of all identified papers to determine if they do not fulfill one or more of the inclusion criteria and, thus, can be excluded. The remaining included articles resulting from this selection were assessed, again, by two reviewers based on the full-text of the articles to see which articles fulfilled all inclusion criteria. Any disagreement on the inclusion was resolved by discussion, or, if no consensus could be reached, a third reviewer was consulted. All duplicate studies were removed. Similarly, two reviewers independently extracted the following data from the included papers using a structured Excel spreadsheet: Year of study; Country where the study was conducted; Conflicts of interest and funding source; Study setting–e.g. commercial/research; Number of employees; Physico-chemical characteristics of the nanomaterials; Study design; Number of study participants; Possible routes of exposure; Description of hygiene measures taken; Description of surveillance system, including components–biological, clinical; Measures taken–e.g. urine samples; symptom questionnaires; clinical investigations–chest X-ray and spirometry; Frequency of taking measurements; Method of measurement: equipment and procedure, with brief description if available; Healthcare worker responsible for taking measures–e.g. occupational hygienist, nurse, doctor, other; Coverage: proportion of workforce participating in surveillance program; Description of action levels–e.g. if symptoms present, if metals in urine etc; Description of how surveillance/screening information is used and any information on the cost of surveillance (program costs) and any confounders. After the data had been extracted, it was compared and where disagreement occurred, it was resolved by discussion and referral to the original text.

Risk of bias for each study was assessed. Given the diversity of study designs included we could not use an existing tool for assessing risk of bias. We constructed the following list of four items, each assessed as low, high or unclear, that we considered the most important risks of bias for studies of medical surveillance or screening of workers exposed to nano-materials:

1. The study used a longitudinal and experimental design (yes = low risk).
2. The study assessed the exposure to nanomaterials with an appropriate measurement technique (yes = low risk).
3. The study used a validated instrument or technique to assess adverse health indicators as indicated by a reference to the validation study or underpinned with data (yes = low risk).
4. For chronic health effects, the study included participants that were exposed long enough/high enough to have a biological effect (> one year = yes, low risk).

A study was considered to have high risk of bias if it met the high risk category in any of these four items. Study heterogeneity was assessed based on ENM group and categories of outcome measures. ENMs groups: metal, metal oxides and organic were considered as similar. Health problems related to organ system were considered similar: cardiovascular and respiratory. Finally indicators of oxidative stress were also considered similar. Since the participants, exposures and outcomes were all diverse, none of the studies could be combined and the results are reported in a narrative review format.

## Results

**Fig 1:**
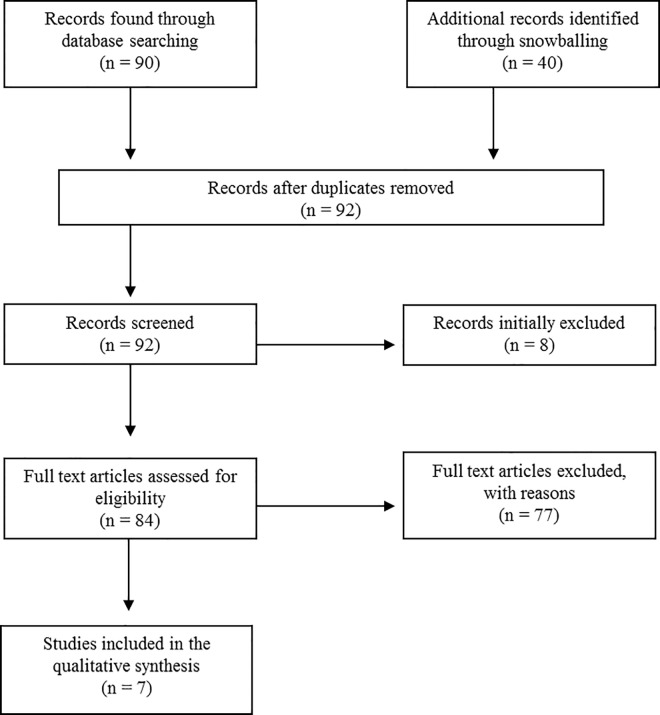
Study overview of included and excluded papers. Refer to S1 Table for exclusion reasons per citation. Note: One study was excluded because full text was not available [24].

A total of 90 citations were retrieved from the initial search as described above. An additional 40 citations were identified through the review of the bibliographies of the initial 90 unique citations. Of these 130 citations, 38 were excluded as duplicates. Thus a total of 92 references were screened for inclusion in the review (Fig 1). Of these, 8 were excluded based on title and abstract. The exact reason for exclusion from further review for each of these publications is tabulated in S1 Table. The remaining 84 citations were reviewed on full-text to assess eligibility. Of these, 77 were excluded. Thus, a total of 7 studies were included [16–23].

A total of 46 studies were excluded because they did not assess or describe a surveillance or screening program in a nano-exposed worker population. In addition, 26 studies were excluded as they did not report specific measures for biological or clinical monitoring. Four studies were excluded because they did not provide sufficient information to identify the nanomaterials workers were exposed to (n = 2) or did not provide information on the methods used to take systematic measurements for the surveillance purposes (n = 2). See Table 1 for details on the characteristics of included studies.

**Table 1:** Description of included studies.

Study	Gause 2011 [16]	Lee 2012 [17]	Liou 2012 [18]	Liao 2014a [19]	Liao 2014b [20]	Lee 2014 [21]	Pelclova 2015 [22,23]
Design type, e.g. descriptive, cross-sectional, longitudinal, exposure case control study	Descriptive Program	Descriptive Program Case Study	Cross-sectional; Exp case control study	Longitudinal (Six-month follow-up) study	Cross-sectional; Exp case control study	Cross-sectional; Exp case control study	Exp case control study (pre- & post-shift exposure)
Country	USA	South Korea	Taiwan	Taiwan	Taiwan	South Korea	Czech Republic
**Participants**							
Occupation or Industry	Commercial research laboratory	Commercial, synthesis	Commercial, 14 plants handling ENMs	Commercial, 14 plants handling ENMs	Commercial, 14 factories handling ENMs	Commercial, manufactures MWCNTs	Commercial TiO_2_ pigment production plant
Participants’ N	N = 200, of which ± 20% exposed	N = 2, both exposed	N = 227 exp; N = 137 unexp	N = 124 expN = 77 unexp	N = 258 expo N = 200 unexp	N = 9 exp N = 4 unexp	N = 36 exp N = 45 unexp
Participants’ age (years)	NS	37 and 42	Exp < 40:173; Exp ≥ 40:54; Unexp < 40:89; Unexp ≥ 40:48 (p = 0.03)	Exp < 40:87Exp ≥ 40:37Unexp < 40:46Unexp ≥ 40:31(p = 0.26)	Exp < 40:194 Exp ≥ 40:64 Unexp < 40:135 Unexp ≥ 40:65 (p = 0.12)	Exp: 33.8 ± 4.9 Unexp: 28.3 ± 4.4	33.5 to 35.0
Participants’ gender (%)	NS	Male exp: 100%	Male exp: 78% Male unexp: 54%	Male exp: 70.8–78% Male unexp: 46.8%	Male exp: 74.8–78.2% Male unexp: 60%	Male exp: 100% Male unexp: 75%	Male exp: 100%
**Medical Surveillance**							
ENM Exposure material type	Carbon-based; Trimetaspheres ENMs; Variety of other ENMs	Metal (nano-Ag)	Metal (nano-Ag, nano-Au); Metal-oxide (TiO_2_, SiO_2_,Al_2_O_3_); Carbon-based (CNT); Nanoresins; Nanoclay; Mixed materials	Metal (nano-Ag);Metal-oxide (TiO_2_, SiO_2_); Carbon-based (CNT)	Metal (nano-Ag, nano-Au);Metal-oxide (Fe_2_O_3_, TiO_2_, SiO_2_, Al_2_O_3_); Carbon-based (CNT); Nanoresins; Nanoclay	Carbon-based (MWCNTs)	Metal-oxide (TiO_2_)
Exposure duration and frequency	NS	7 years	Duration: 2.87 ± 2.34 years; Frequency: 2.43 times/week, 2.69 h/time; Exposure time: 7.91 h; Total cumulative exposure: 2,260 h	At baseline: Duration: 3.22 years; Frequency:2.43 times/week, 2.69 h/time; Exposure time: 8 h. After 6 months of NM exposure: Frequency: 2.78 times/week, 2.79 h/time; Exposure time: 9.38 h	Duration: 0.1 to 7 h/time; Frequency: 0.33 to 12 times/week	3.9 ± 3.9 years, with 8 h shifts	3.8 to 9.7 years, with 2.5 to 5.5 h per shift
Exposure route, e.g. inhalation / ingestion / dermal contact / *in vivo* administration	NS	Inhalation	Potential inhalation and/or dermal contact	Potential inhalation and/or dermal contact	Inhalation for powders; Dermal contact for gel & liquid solutions	Inhalation	Inhalation
Monitoring of exposure quantity, e.g. personal, internal or environmental	Environmental (amount not specified)	Personal (0.35 and 1.35 μg/m^3^ nano-Ag per 0.158 and 0.109 mg/m^3^ of TSP, respectively)	Self-administered exposure questionnaire identified potential inhalation and/or dermal contact	Environmental (amount used/manufacturing), with ranges between 2.1 to 4,000,000 mg/time	Environmental (amount used/manufacturing), with ranges between 2.1 to 4,000,000 mg/time	Personal and environmental, where elemental carbon was 6.2–9.3 μg/m^3^ in personal sampling and 5.5–7.3 μg/m^3^ in area sampling	Internal (EBC), where total mass of TiO_2_ concentrations were between 0.40 and 0.65 mg/m^3^
Type of study, e.g. Biomarker–based / Clinical	Clinical	Biomarkers / Clinical	Biomarkers / Clinical	Biomarkers / clinical	Clinical	Biomarkers/Clinical	Biomarkers /Clinical:
Target	General Health	Blood and urine nano-Ag concentrations; Blood chemistry and FBC	Lung inflammation; Oxidative damage; Antioxidant enzymes; Cardiovascular markers; Genotoxicity; Lung function; Neurobehavioral function	Antioxidant enzymes; Lung inflammation; Cardiovascular markers; Genotoxicity; Lung function; Neurobehavioral function	Cardiovascular and respiratory symptoms/diseases; Sneezing; Skin diseases; Neurological symptoms	Health effects, e.g.: Pulmonary function test and lung response for inflammatory, oxidative stress and lipid peroxidation; Blood metal concentration; Blood chemistry and FBC	Lung Function; Pulmonary oxidative stress markers
Indicators& Biomarkers	“Periodic health status”; Lung function; Chest X-Ray	Nano-Ag concentration in blood and urine; Blood chemistry and FBC	Blood, urine and EBC used for antioxidant enzymes (MPO,GPX-1, SOD); Lung inflammation and oxidative damage markers (CC16, HSP 70, NO, NF-ΚB, 8-oxodG, m7G, 8-iso-PGF2α); Cardiovascular biomarkers (fibrinogen, VCAM, ICAM-1, IL-6, IL-6sR, arylesterase, paraoxonase, CRP); heart rate variability; Genotoxicity biomarkers (comet assay, MN); Lung function (FVC, FEV1, PEFR,MMF,FEF25%, FEF50%,FEF75%); Neurobehavioral tests (reaction time, memory tests).	Blood, urine and EBC used for antioxidant enzymes (SOD, GPX-1);Lung inflammation and oxidative damage markers (CC16, NO, NF-ΚB, 8-oxodG, m7G, 8-iso-PGF2α); Cardiovascular biomarkers (fibrinogen, VCAM, ICAM-1, IL-6,IL-6sR,arylesterase, paraoxonase, CRP, MPO);Heart rate variability; Genotoxicity biomarkers (comet assay, MN); Lung function (FVC, FEV1, PEFR, MMF, FEF25%, FEF50%, FEF75%); Neurobehavioral tests (reaction time, memory tests)	Self-administered symptom questionnaire, for “Work-relatedness of symptoms”, including sneezing and nose obstructions, difficulty breathing, chest pain, sweating, nausea/vomiting, dizziness, Hyperlipidemia, skin irritations and other potential diseases (atopic dermatitis, allergic dermatitis, skin cancer, Arrhythmia, Ischemic heart disease, Angina Valve heart disease)	The PFT as a percentage of FEV1, FVC; EBC oxidative stress biomarkers (MDA, H_2_O_2_, 4-HHE, n-hexanal); Blood content of catalyst metals may act as surrogate markers for MWCNT exposure, i.e. concentration of Co and Mo	Ti concentration in EBC; Markers of oxidation of nucleic acids (8-OHdG, 8-OHG, 5-OHMeU) and proteins (o-Tyr, 3-Cl-Tyr, 3-NOTyr) in EBC.
**Program Outcomes**							
Summarized result	N/A	Nano-Ag in blood and urine not elevated (Blood nano-Ag concentration: 0.034 and 0.030 μg/dl; Urine nano-Ag concentration: 0.043 μg/dl and not detected)	No significant difference in lung inflammation, oxidative damage, genotoxicity or pulmonary function; Antioxidant enzymes decreased (SOD & GPX-1); Increased cardiovascular markers (fibrinogen, ICAM, IL-6)	Cardiovascular injury(increase in VCAM and decrease in paraoxonase); Pulmonary injury, i.e. a decrease in CC16 and lung function, i.e. not observed in previous study [18]; No significant change in heart rate, nor increase in oxidative stress or lipid peroxidation markers, only adecrease of SOD and GPX-1 was observed.	The study was limited to self-reported evaluations to heterogenous ENMs, which were compared to control banding tools that require validation.	Pulmonary stress increased, where MDA, 4-HHE and n-hexanal levels were higher; Significant difference in Co or Mo.	All oxidative markers were statistically significantly elevated in the exposed vs. the unexposed group
Early signs	N/A	Routine blood chemistry and FBC not deviant	1 out of 3 neuro-tests impaired; Lung function showed no impairment	Lung impairment based on FVC, FEV1, FEF50%, FEF75%	N/A	Routine blood chemistry and FBC not deviant; Increased FEV1 in exposed workers, normal ratios	Lung function showed no impairment
Clinical signs	N/A	N/A	N/A	N/A	Increased prevalence of allergic dermatitis and ‘sneezing’	N/A	N/A
Interpretation of outcomes and proposed recommendations	Nano-specific surveillance not deemed necessary	Surveillance measures did not detect any abnormalities	Although some biomarkers were elevated, these markers are not specific for ENM exposure	Although some biomarkers were elevated, these markers are not specific for ENM exposure	Allergic dermatitis has multiple causes, not specific for ENM exposure, where “Sneezing” is a non-specific symptom.	Oxidative stress markers increased, but the study had a small sample size and non-specific markers. MDA, 4-HHE and blood Mo content are proposed as useful biomarkers for MWCNTs.	Oxidative stress markers in EBC were significantly elevated in exposed group; Lung function tests were normal
**Costs & Coverage**							
Monetary / Resource use	NS	NS	NS	NS	NS	NS	NS
Coverage	NS	2 out of 5 workers participated	97% of workers participated	Follow-up rate of 67.2%	NS	NS	NS

3-Cl-Tyr = 3-chlorotyrosine; 3-NOTyr = 3-nitrotyrosine; 4-HHE = 4-hydroxy-2-hexenal; 5-OHMeU = 5-hydroxymethyl uracil; 8-iso-PGF2α = 8-iso-prostaglandin F2α; 8-OHdG = 8-hydroxy-2-deoxyguanosine; 8-OHG = 8-hydroxyguanosine; 8-oxodG = 8-Hydroxydeoxyguanosine; Ag = Silver; Al_2_O_3_ = Aluminium oxide; Au = Gold; CC16 = Clara cell protein; CNT = Carbon nanotubes; Co = Cobalt; CRP = C-reactive protein; EBC = Exhaled breath condensate; ENM = Engineered nanomaterial; Exp = Exposed; FBC = Full Blood Count; Fe_2_O_3_ = Iron oxide; FEF25% = Forced expiratory flow at 25%; FEF50% = Forced expiratory flow at 50%; FEF75% = Forced expiratory flow at 75%; FEV1 = Forced expiratory volume in 1 second; FVC = Forced vital capacity; GPX-1 = Glutathione peroxidase 1; H_2_O_2_ = Hydrogen peroxide; HSP 70 = Heat shock protein 70; ICAM-1 = Intercellular adhesion molecule 1; IL = Interleukin; IL-6 = Interleukin-6; IL-6sR = Interleukin-6soluble receptor; m7G = 7-Methylguanosine; MDA = Malondialdehyde; MMF = Maximal midexpiratory flow; MN = Micronucleus;Mo = Molybdenum; MPO = Myeloperoxidase; MWCNT = Multi-walled carbonnanotubes; N/A = Not applicable; NF-ΚB = Nuclear factor-κB; NO = Nitric oxide; NS = Not stated; o-Tyr = o-tyrosine; PEFR = Peak expiratory flow rate; PFT = Pulmonary function test; PG = Prostaglandin; SiO_2_ = Silicon dioxide; SOD = Superoxide dismutase; Ti = Titanium; TSP = Total suspended particulate;Unexpo = Unexposed; VCAM = Vascular cell adhesion molecule.

### Study designs

The published designs ranged from case reports to cross-sectional assessments and did not follow a set format providing the minimum required information on ENM characterisation, the type of exposure and the measured indicators/biomarkers. The study by Gause *et al*. [16] reported a surveillance program in a small business in the USA with approximately 200 workers of which 20% were exposed to ENMs. One study initially reported findings from 2012 [22], with 20 exposed workers (16 in production and 4 in research positions) and 20 control participants, as well as, findings from 2013, with 28 exposed workers (14 in production and 14 in office positions) and 25 control participants [22]. Subsequently, an update of this study [23] reported 36 exposed workers (32 in production and 4 in research positions) and 45 control participants. All available data from both reports are included in Table 1. The remaining five studies [17–21] included a total of 1078 participants. All the included studies were heterogeneous in terms of study design, design of the surveillance system as well as types of ENMs workers were exposed to. Therefore, this review does not lend itself to pooling results for intensive analyses and the results were reported in a narrative. One study was a case study [17], three were cross-sectional in design [18,20,21], one was stated as longitudinal, but was a follow-up cross sectional study [19] and one study was a case study that reported results pre- and post-shift exposure [23]. One study was conducted in Virginia, United States [16], one in the Czech Republic [23], three in Taiwan [18,19,20] and two in South Korea [17,21].

### Types of participants and exposures

The case study by Lee *et al*. [17] included two male participants aged 37 and 42. Both had a total of seven years’ occupational history of exposure to silver nanomaterials through their work at a silver nanoparticles production plant producing ~5kg/day. Closed system engineering controls were in place.

The second study included 13 workers, of which four were office workers (1 female; 3 males) with a mean age of 28.3 years (SD ± 4.4) and nine workers (all male) with a mean age of 33.8 years (SD ± 4.9) by Lee *et al*. [21]. The workers were involved with the manufacturing of MWCNTs. The exposed workers had an average of 3.9 years (SD ± 3.9, median 2 years) occupational history of exposure.

After conducting a national survey of nano-producing factories in Taiwan, Liou *et al*. [18] recruited 364 workers from 14 different ENMs handling plants in Taiwan. Of the exposed workers (n = 227), defined as workers either directly or indirectly handling ENMs, 177 were male and 50 were female. For the non-exposed group (n = 137) 74 were male and 63 were female. A statistically significant difference in age was found between exposed and unexposed workers with unexposed workers being older than exposed workers (p = 0.03). A six-month follow up study was also conducted and reported by Liao *et al*. 2014a [19] on the same participants. Of the exposed workers (n = 124), 92 were male and 32 were female. For the non-exposed group (n = 77), 36 were male and 41 were female. A statistically significant difference in gender was found between exposed and unexposed workers (p < 0.01). In addition, the higher risk group consisted of more men who had a higher education, whilst the control group consisted of women with a university education.

In the next study, Liao *et al*., 2014b [20] conducted a cross sectional comparison of symptoms between 258 nano-exposed workers (197 male, 61 female) and 200 unexposed workers (120 male, 80 female) using a self-administered questionnaire. There was no statistically significant difference in the age of unexposed and exposed workers (p = 0.12).

Although not explicitly stated in either study, it is suggested that the Liou *et al*. [18] and Liao *et al*., 2014b [20] studies were conducted on the same participants. Both studies were conducted in Taiwan by the same group of authors, both recruited participants from 14 different nanomaterial handling plants and collected the same variables for analysis. Further the follow-up study by Liao *et al*., 2014a [19] was also conducted on the same study population. Due to the different settings from which workers were recruited for these studies, exposure to ENMs were heterogeneous and included exposure to metal, metal oxide and carbon based ENMs. Exposed participants were further stratified into two risk groups in both studies. Risk stratification was done following the control banding approach developed by Paik *et al*. [25] and was a function of the severity score of the ENMs toxicity and the score of the exposure probability. Risk group 1 was considered the lower risk group and was composed of 139 participants (35 female, 104 male) in the Liao *et al*., 2014b [20] study and 128 (31 female, 97 male) in the Liou *et al*. [18] study. Risk group 2, the higher risk group, had 119 participants (26 female, 93 male) in the Liao *et al*., 2014b [20] study and 99 participants (19 female, 80 male) in the Liou *et al*. [18] study.

Pelclova *et al*. [22] conducted a pre- and post-shift study on 20 workers exposed to TiO_2_ aerosols and 20 unexposed workers in the Czech Republic. The initial measurements were taken in 2012 with repeat measures taken a year later in 2013. Initially, only an abstract detailing the findings from this study was available and no demographic information on the participants were reported. Since then, an update has been published that reports 36 male workers participated in the study [23].

### Types of interventions

#### Biomarkers

Analysis of exhaled breath condensate (EBC) was performed in four studies [16, 18, 19, 21–23]. Lee *et al*. [21] assessed differences between workers exposed to MWCNTs on pulmonary response by measuring non-invasive stress biomarkers (malondialdehyde, H_2_O_2_, 4-HHE and n-hexanal), which were collected after workers’ shifts. Similarly, Liou *et al*. [18] and Liao *et al*., 2014a [19] assessed the difference between workers exposed to a heterogeneous mix of ENMs in markers of lung inflammation (Clara cell protein, serum heat shock protein 70, serum nuclear factor-kB transcription factor activation, nuclear factor-kB transcription factor activation and nitric oxide) as well as markers of oxidative damage (urine 8-hyroxydeoxyguanosine; methyl guanosine; plasma 8-hyroxydeoxyguanosine and isoprostane). Pelcolva *et al*. [22] analyzed differences in Malondialdehyde, 4-HHE, 4-HNE, 8-isoProstaglandin F2α and aldehydes from EBC between workers exposed to TiO_2_ and unexposed workers before and after the commencement of a shift. These results were refined and reported as differences in nucleic acids (8-OHdG, 8-OHG, 5-OHMeU) and proteins (o-Tyr, 3-ClTyr, 3-NOTyr) [23].

Routine blood chemistry and full blood counts (FBC) were performed on participants in two studies [17, 21]. In one of these studies participants were exposed to nano-Ag and urine and blood silver concentrates were also measured [17]. In the second study participants were exposed to MWCNTs and, because metals are utilized in the synthesis of these ENMs, blood cobalt and molybdenum were measured [21]. Antioxidant enzyme activity (superoxide dismutase, glutathione peroxidase and myeloperoxidase) and cardiovascular biomarkers (fibrinogen; intracellular adhesion molecule-1; and interleukin-6) were also assessed in two studies [18, 19].

#### Clinical monitoring

Pulmonary function tests were conducted on participants in four studies [18, 19, 21, 22, 23]. The surveillance program described by Gause *et al*. [16] also undertook baseline and periodic lung function tests as well as chest X-rays on all at-risk workers [5]. Liou *et al*. [18] also conducted neurobehavioral tests on participants to assess reaction time and memory. A self-administered symptom questionnaire was utilized in one study to assess the prevalence of cardiovascular, respiratory, skin and neurological symptoms and diseases between exposed and unexposed workers. The work-relatedness of symptoms were also assessed through the questionnaire by asking whether or not the symptom was present or not present before being exposed to ENMs [20].

#### Other interventions

Genotoxicity was assessed in two studies [18, 19]. The surveillance program described by Gause *et al*. [16] included detailed worker record keeping, characterization of baseline and periodic health status of workers (including lung function test and chest X-ray) and maintaining a log-book of exposure activities. Given the low exposure risk, the workplace did not have a non-specific medical surveillance program. However, this was re-evaluated through regular risk assessment.

### Findings of the medical surveillance programs

#### Primary outcomes

*Biomarkers*. EBC concentrations of malondialdehyde, 4-HHE and n-hexanal/aldehyde were statistically significantly different between unexposed workers and exposed workers with all three being elevated in the exposed group in two studies [21, 22, 23]. Notwithstanding the measurement of different markers, Liou *et al*. [18] found no statistically significant difference between unexposed and exposed workers for markers of lung inflammation (Clara cell protein, serum heat shock protein 70, serum nuclear factor-κB transcription factor activation, nuclear factor-κB transcription factor activation, nitric oxide) or markers of oxidative damage (urine 8-hyroxydeoxyguanosine; methyl guanosine; plasma 8-hyroxydeoxyguanosine and isoprostane) in EBC. However, cardiovascular biomarkers (fibrinogen; intracellular adhesion molecule-1; and interleukin-6) were significantly increased in the exposed group when compared to controls. The antioxidant enzymes superoxide dismutase and glutathione peroxidase’s activity were both significantly decreased in the exposed group.

Repeat measures of the same biomarkers on the same study populations six months later [19] found significant reduction in serum Clara cell protein in the exposed group, with no difference in any of the other lung inflammation or oxidative damage markers. A significant decrease in the cardiovascular biomarker, paraoxanase was observed in the exposed group as well as a significant increase in the VCAM cardiovascular biomarker. No other cardiovascular biomarkers changes significantly during the six month follow-up. Lastly, a significant decrease in antioxidant activity (SOD and GPX-1) was observed between baseline and follow-up in the exposed group.

In the Lee *et al*. [17] case study, neither blood cobalt nor molybdenum was significantly different between the exposed and unexposed groups and the urine and blood silver concentrations were not elevated in either of the workers examined, i.e. the blood silver in the first participant was 0.034 μg/dl and in the second participant 0.030 μg/dl, whereas the urine silver in first participant was 0.043 μg/dl and was undetectable in the second participant. Routine blood chemistry and blood counts were normal with no differences between exposed and unexposed groups.

*Clinical monitoring and other findings*. No clinically or statistically significant difference on any of the lung function parameters were found between exposed and unexposed workers [18, 23]. Liao *et al*., 2014a [19] reported a statistically significant reduction in three lung function parameters (maximal mid-expiratory flow, peak expiratory flow and forced expiratory flow) among exposed workers on repeat measures six-months after baseline lung function measures were taken. No statistically significant reduction in any of the lung function parameters of unexposed workers was reported. The analysis was adjusted for smoking status in both the Liou *et al*. [18] and Liao *et al*. [19] studies. The neurobehavioral tests that showed “correct rate of 7-digit backward memory” was significantly lower in the exposed group compared to the unexposed group, however reaction time and correct rate of 7-digit forward memory was not [18]. There was no significant difference between the genotoxicity results between the exposed and unexposed groups [18, 19].

The only symptom, which was found to be significantly more prevalent among exposed workers, was sneezing (5.88% prevalence in risk level 2 and 7.91% in risk level 1 vs. 2.00% in controls, p = 0.04) [20]. However, the self-administered symptom questionnaire reported symptoms related to other nose obstructions (Rhinitis, Rhinorrhea), as well as, difficulty with breathing (a dry cough, productive cough, wheezing, shortness of breath), chest pain (tightness, oppression, pain with radiation to left arm/shoulder/chin/back, irritable in addition to pain, burning sensation, unexpected pain without resolve after 10 to 15 min rest, stroke), sweating, nausea/vomiting, dizziness and Hyperlipidemia. The only disease, which was significantly worsened by handling ENM, was found to be allergic dermatitis (4.20% prevalence in risk group 1 and 0% in risk group 2 versus 0.5% in the control group). However, the self-administered symptom questionnaire reported symptoms related to other skin irritations (Folliculitis, pigmentation). This was in addition to other potential diseases, e.g. Atopic dermatitis, skin cancer; Chronic Bronchitis, Emphysema, Asthma, Tuberculosis, lung cancer, Arrhythmia, Ischemic heart disease, Angina Valve heart disease.

#### Secondary outcomes

None of the included studies reported on any cost information. Four studies did not report any coverage information [16, 20, 21, 23]. A 97% participation rate was reported in Liou *et al*. [18], whereas two out of a potential five exposed workers were reported to participate in Lee *et al*. [17]. Liao *et al*. [19] reported a follow-up coverage rate of 67.2%.

### Risk of bias in included studies

There was significant risk in bias in all the included studies. Table 2 below provides a summary of study bias. An explanation of the bias is provided in the supplementary information, e.g. publication bias or selective reporting within studies (Materials and Methods, S2 Text and S2 Table).

**Table 2:** Summary of study bias.

Study	Gause *et al*., 2011 [16]	Lee *et al*., 2012 [17]	Liou *et al*., 2012 [18]	Liao *et al*., 2014a [19]	Liao *et al*., 2014b [20]	Lee *et al*., 2014 [21]	Pelclova *et al*., 2012 [23]
Design	High	High	High	High	High	High	High
Exposure Measure Technique	Unclear	Low	High	High	High	Low	Low
Measure of health indicator	High	Low	Low	Low	High	Low	Low
Exposure duration	Unclear	Low	Unclear	Unclear	Unclear	Low	Low

## Discussion

A number of screening and surveillance approaches specific to workers exposed to ENMs were found. However, the evidence to support the utilization of medical screening or surveillance programs, specifically directed at the early identification of adverse health effects related to exposure to ENMs, was found to be limited and of low quality. Underlying all the problems associated with establishing any screening and medical surveillance programs is the bigger issue related to measuring the actual ENM exposure. The constraints observed when performing exposure assessments for different kinds of materials and different exposure scenarios have already been reviewed elsewhere [26]. However, examples of these problems include the metrics of nanoparticle assessment and lack of equipment personnel sampling [27]; a shortage of equipment and methods that are suitable for environmental sampling, where measurements are able to differentiate between background from incidental exposure [27, 28]; surface area measurement analysis of nanoparticles e.g. for CNTs and fibre counts [18]. The greatest obstacle is a lack of summary indices for heterogeneous nanomaterial exposure, where testing workers without known exposure measurements, or, without predictive values for positive and negative test results means that the information cannot be reported with any true significance. It could be argued that if this data is not currently available, then why bother to develop a medical surveillance program at all? Perhaps the prudent answer is to err on the side of caution in order to protect workers, even with the limited equipment and information currently available. On the other hand, one should also consider the false reassurance that “testing is better than not testing”. Further, there is no information on the cost of screening, resource use or user satisfaction, in addition to very little information on user acceptability as measured by participation rate or coverage.

Currently, another key limitation to the design and implementation of ENM-specific workplace screening and surveillance programs is the lack of validated biomarkers for nanomaterial exposure. Even though some of the studies found an elevation of biomarkers in exposed workers, it is impossible to say if this due to the exposure. A secondary problem is the lack of information on the cost-effectiveness and acceptability of candidate biomarkers, as well as, screening approaches in identifying at risk workers at an early stage and improving health outcomes. These limitations are addressed in the literature with a number of authors concluding that currently there is insufficient scientific evidence to recommend targeted medical screening for workers potentially exposed to most ENMs [29–31]. In addition, although currently available medical tests have not been validated for use in asymptomatic workers exposed to ENMs [30,31], based on other types of data (including animal studies and health effects from exposure to macro-forms of relevant agents), NIOSH has recommended medical screening for workers exposed to CNTs [3]. Given these limitations, careful consideration should be given to establishing routine occupational health surveillance for workers exposed to ENMs.

Utilizing data collected from medical surveillance programs, however, could provide evidence on health outcomes associated with various ENMs exposures and as such, inform early intervention, i.e. improve controls and decrease exposure [14, 30–32]. Nano-specific medical screening and surveillance programs must be based on a thorough needs assessments [30,31], which will require periodic modification as more evidence becomes available [33]. Screening remains a form of secondary prevention and the first priority should be to implement appropriate controls to decrease exposure [30,31].

The specific effects of nanomaterials (especially poorly soluble or insoluble ones) may be so-called “particle” effects and are, thus, not related to their chemical composition. Therefore, their expected health effects may resemble those associated with pollution-related ultrafine particles [34] and parallels can be drawn between the health effects of nanomaterials and ultrafine particles or bulk materials, e.g. carbon black and amorphous silica [35]. Indeed, the French Institute for Public Health Surveillance (*Institut de Veille Sanitaire*, *InVS*), under the guidance of the French Ministries of Health and of Labour, is establishing an epidemiological surveillance system of workers likely to be exposed to ENMs based not only on CNT and TiO_2_, but also on existing information gathered from carbon black and amorphous silica. An initial step in establishing an epidemiological surveillance system to identify health outcomes associated with specific exposure to nanomaterials requires establishing a registry of exposed workers and linking the exposure data to routine occupational health surveillance data [35]. This could be feasible depending on the level of sophistication of existing health information systems. Basic national exposure registries can be established with limited resources by identifying workplaces involved with the synthesis or application of ENMs. These registries can form the basis for longitudinal exposure data collection and, when combined with health surveillance, the identification of health outcomes associated with nanomaterial exposure.

A recent review of available epidemiological evidence by Liou *et al*. [36] also found insufficient evidence to link health outcomes with workplace exposure to ENMs. The review included five of the studies that are included in our review [17–19, 21, 23], but found an additional eight unpublished studies and one published study we did not identify [37]. The unpublished studies were, however, mainly the review authors’ own studies and would have been difficult to locate without prior knowledge, thus, these did not comply with the unbiased inclusion criteria implemented herein. Liou *et al*. [36], in their review, conclude that the studies that they found form the first wave of epidemiological studies on nanomaterial workers. They also acknowledge that it is impossible to conclude if the health effects found are due to the exposure to nanomaterials. This is especially due to the cross-sectional nature of the studies and the lack of a clear, generally accepted, exposure definition. The authors are probably correct in predicting that more studies on health effects of nanomaterials will be published in the near future, hence updated systematic reviews will also be required.

The study herein undertook an extensive database and web-based search strategy to locate all available studies. However, it is difficult to develop an exhaustive search strategy for nanomaterials since not all authors describe the material in terms of “nano”, but could for example use the term “ultrafine”. In addition, a number of nanomaterials have specific names, e.g. fullerene. However, a number of additional studies were found through snowballing. Despite the use of this search strategy being much more comprehensive than the one previously used by Liou *et al*. [36], only one additional study was found [16]. It should also be emphasized that the search strategy focused on surveillance programs. Therefore, the occupation of the worker was not essential to this review, but rather that the worker in whatever capacity was at risk of exposure to ENMs. It could be argued that specific occupations, e.g. “health care providers” (referring to the biomedical applications of ENMs), were not well represented by using the search terms “health examination”, “health surveillance”, “physical examination”, “medical”, “medical surveillance”, “occupation” and/or “work”. However, the focus of this review was to determine the latest findings from surveillance programs that could inform and protect workers, i.e. not an exhaustive list of all occupations that are at risk of exposure to ENMs. Other limitations to this review, which can be interpreted as recommendations for future work, include:

1. The differences and variety of the published designs, which ranged from case reports to cross-sectional assessments, where different types of information were provided, with subsequent differences in scientific inference.
2. The data extracted that was obtained from testing workers without known exposure measurements, nor established predictive values for positive and negative test results, with subsequent poor significance.

Irrespective of the listed limitations, this systematic review is still relevant because it proves that, although surveillance programs have been initiated and preliminary results are being published, the current studies are actually not answering the important questions or solving the overall problem regarding what the potential health hazards are among workers, i.e. those either handing ENMs or those potentially exposed to ENMs. Hence, there is insufficient evidence to recommend a targeted nano-specific workplace medical screening or surveillance programme for most ENMs. However, it is recommended that both exposure surveillance and routine medical screening be strengthened by reporting using a set format of minimum required information as previously suggested [28,38]. In addition, where feasible, a registry should be established. It should be noted that this registry will only be useful if it is applied in parallel with exposure measurement data (which is currently also lacking). This then implies that better reporting of the exposures is recommended, e.g. the type and quantity of exposure, with an emphasis on the exposure measurement method used. It is also recommended that additional research be performed on the validation of candidate biomarkers as measures for the early detection of adverse health outcomes associated with exposure to ENMs. It was also noted that the surveillance programs did not focus on waste disposal of ENMs or the GLP-based decontamination of equipment in contact with ENMs and, as such, is now another recommendation. Finally, there should be increased efforts to raise awareness of all workers from all applications, whether it may be bio-medical, optical or electronic fields regarding the possible risk of exposure, in order to gain support and willing participation in future proposed surveillance programs at the workplace.

## Supporting Information

S1 Table List of excluded studies with reason for exclusion. (DOCX)

S2 Table PRISMA 2009 checklist. (DOC)

S1 Text Development of Systematic Evidence Review for WHO Guidelines on Protecting Workers from Potential Risks of manufactured Nanomaterials. (DOC)

S2 Text Search strategy. (DOC)
