# Supplementary material for: Systematic Review of Screening and Surveillance Programs to Protect Workers from Nanomaterials
Source: PLoS One. 2016 Nov 9;11(11):e0166071. doi: 10.1371/journal.pone.0166071 (PMC5102462; doi:10.1371/journal.pone.0166071)
Supplement: S1 Table — (DOCX) [file pone.0166071.s001.docx]

**Supplementary Information**

**List of Excluded studies with reason for exclusion**

| **Number of articles excluded** | **Exclusion Criteria** | **Reference** |
| --- | --- | --- |
| 46 | Does not assess or describe the systematic collection and/or analysis of NM-specific occupational health and safety information which aims to identify exposure to NMs or to monitor health status of workers potentially exposed to NMs in the work place | ACOEM (2011); Anderson(2013); Andrews(2012); Armitage (1996); Aschberger (2011); Azqueta (2009); Barkalina(2011); BASF (2004); Beramaschi(2009); Beramaschi(2015); Bertinetti (2009); Castranova (2011); Collins(2012); Collins(2014); Du (2011); EUROTOX(2012); Faist (2012); Franco (2011); Friedrichs (2007); Gorner(2015); Handy (2007); Harber (2003); Iavicoli (2014) Li (2011); Li (2009); Li(2014); Madl (2009); Methner (2009b); Nasterlack (2008); Nasterlack (2011); Pachman (2009); Pilehvar (2014); Qian(2013); Ross(2009); Serra (2007); Sigolaeva (2010); Song(2010); Stone(2010); Teixeira(2012a); Wattamwar(2011); Weissman(2014); Weng(2013); Yokel(2011); Zober (1996); Zou (2010) |
| 26 | Does not report the internal (biological monitoring) and/or clinical signs and symptoms (clinical monitoring) of workers involved with the synthesis and/or application and/or handling of any NMs for commercial or research purposes | Birch (2011); Boutou-Kempf (2011); Fischman (2011); Han (2008); Methner (2009a); NIOSH (2009); Paik (2008); Ponce(2013); Riediker (2012); Roisman (2011); Rossi(2010); Sayre (2011); Schulte (2008); Schulte (2008); Schulte (2008a); Schulte (2008b); Schulte (2009); Schulte (2011); Schulte (2012); Schulte(2014); Simonsen (2012); Sng (2011); Trout (2010); Trout (2011); Wolinsky (2006); Zalk (2009) |
| 7 | Excluded based on title and abstract | Chiung (2009); Pena (2011); Selden (1993); Stelting (2005a); Stelting (2005b); Teixeira (2012b); Woskie (2010) |
| 2 | Does not provide sufficient information to identify the NMs workers are exposed to | Satta (2011); Sauvain (2014) |
| 2 | Does not report the internal (biological monitoring) and/or clinical signs and symptoms (clinical monitoring) of workers involved with the synthesis and/or application and/or handling of any NMs for commercial or research purposes | Boutou-Kempf (2011); Guseva (2013) |
| 1 | Full text not available | Stebounova (2012) |
