# Supplementary material for: Systematic Review of Screening and Surveillance Programs to Protect Workers from Nanomaterials
Source: PLoS One. 2016 Nov 9;11(11):e0166071. doi: 10.1371/journal.pone.0166071 (PMC5102462; doi:10.1371/journal.pone.0166071)
Supplement: S1 Text — (DOC) [file pone.0166071.s003.doc]

| National Institute for occupational health |
| --- |
| **Development of Systematic Evidence Review for WHO Guidelines on Protecting Workers from Potential Risks of manufactured Nanomaterials** |
| *What worker health surveillance approaches, if any, should be implemented for workers at risk from exposure to specific nanomaterials or groups of nanomaterials?* |
|  |
| **Mary Gulumian, Jos Verbeek, Pieter de Jager, Natasha Sanabria, Charlene Andraos** |
|  |

**15 June 2015**

Contents

**List of tables** [**3**](#__RefHeading___Toc292895406)

1. Introduction [4](#__RefHeading___Toc292895407)

2. Aim and objectives [5](#__RefHeading___Toc292895408)

3. Methods [5](#__RefHeading___Toc292895409)

3.1 PICO Question [5](#__RefHeading___Toc292895410)

3.2 Exclusion Criteria [6](#__RefHeading___Toc292895411)

3.3 Inclusion Criteria [6](#__RefHeading___Toc292895412)

3.4 Search strategy [7](#__RefHeading___Toc292895413)

3.5 Study selection [8](#__RefHeading___Toc292895414)

3.6 Data extraction and management [8](#__RefHeading___Toc292895415)

3.7 Assessment of heterogeneity [11](#__RefHeading___Toc292895416)

3.8 Assessment of study quality [12](#__RefHeading___Toc292895417)

4. Time lines and budget [12](#__RefHeading___Toc292895418)

5. References [14](#__RefHeading___Toc292895419)

# List of tables

Table 1: Databases to be search for this systematic review [7](#__RefHeading___Toc292895420)

Table 2: Search strategy for this review [7](#__RefHeading___Toc292895421)

Table 3: Data variables to be extracted [8](#__RefHeading___Toc292895422)

Table 4: Matrix for grouping outcome measures together [11](#__RefHeading___Toc292895423)

# Introduction

The World Health Organization defines public health surveillance as the “…continuous, systematic collection, analysis and interpretation of health-related data needed for the planning, implementation and evaluation of public health practice”.[1] The purpose of a public health surveillance programme is: i) to serve as an early warning system; ii) to monitor the effectiveness of health interventions over time and; iii) to conduct research and inform public health policy.[1]

Occupational health surveillance, similarly has been defined as “…the ongoing systematic collection, analysis and dissemination of exposure and health data on groups of workers for the purpose of early detection of disease and injury”.[2] Occupational health surveillance consist of environmental (exposure) surveillance and medical surveillance, a component of which is medical screening.[3] Medical surveillance involves the continuous systematic collection, analysis and interpretation of specific health events (clinical) and/or changes in/of biological functioning (biological monitoring).

Nanomaterials (NMs) are increasingly produced and utilized in a wide range of products - the health and environmental effects of which remains uncertain.[4] Following the precautionary principle, a component of which includes taking preventive action in the face of uncertainty,[5] workplace surveillance systems for workers exposed to NMs could potentially help early detection of health effects; serve as a source of data to evaluate epidemiological links between exposure and health outcomes; and inform action to prevent disease due to exposure to NMs.

A number of challenges emerge when considering the design of surveillance programmes required for workers who are exposed to NMs. These include the heterogeneity of engineered NMs; a lack in understanding of various exposure routes, absorption, metabolism and excretion of NMs; a poor understanding of the health outcomes associated with exposure to various NMs, as well as, a lack in validated tests to be utilized in biological monitoring for asymptomatic exposed workers.[6] Further to this, it is not clear whether or not the benefits of a targeted surveillance programme for NMs would outweigh the costs. Thus, there are a number of questions concerning the affordability, acceptability and relevance of surveillance systems for engineered NMs in the workplace.

# Aim and objectives

The aim of this systematic review is to identify worker health surveillance approaches which could be implemented for workers at risk from exposure to specific NMs or groups of NMs. The objectives are to:

1. Identify any NMs-specific surveillance programmes
2. Describe all identified NMs-specific surveillance programmes in terms of:
   1. the work setting in which they have been implemented;
   2. the components of the surveillance programme (e.g. biological and/or clinical)
   3. the information collected by the surveillance programme;
   4. the use (and application) of the information;
3. To summarise and provide a synthesis of the available evidence

# Methods

A systematic review will be undertaken to identify worker health surveillance approaches which could be implemented for workers at risk from exposure to specific NMs or groups of NMs.

## PICO Question

**Population:** Workers exposed to any type of manufactured nanomaterial for commercial or research purposes.

**Intervention:** Any systematic collection and/or analysis of NM-specific occupational health and safety information which aims to monitor health status of workers potentially exposed to NM in the work place

**Comparator:** None or other alternative

**Outcome:** Primary: 1. Symptoms, signs, abnormal laboratory or imaging tests detected. 2. ENM /their metabolites or biomarkers in blood or urine as an internal dose of exposure. As a secondary outcome any information on the cost of the surveillance programme and the coverage (defined as the number enrolled in surveillance programme/total number of employees) of the surveillance programme will be captured.

## Exclusion Criteria

The following exclusion criteria will be applied:

1. Studies that do not assess or describe the systematic collection and/or analysis of NM-specific occupational health and safety information which aims to identify exposure to NMs or to monitor health status of workers potentially exposed to NMs in the work place will be excluded;
2. Studies that do not report the internal (biological monitoring) and/or clinical signs and symptoms (clinical monitoring) of workers involved with the synthesis and/or application and/or handling of any NMs for commercial or research purposes, will be excluded;
3. Studies that do not provide sufficient information to identify the NMs workers are exposed to; and
4. Studies that do not provide information on the methods used to take systematic measurements for the surveillance purposes (i.e. biological and clinical measures) will be excluded from the analysis.

## Inclusion Criteria

The following inclusion criteria will be applied:

1. All study designs will be included:
   - descriptive studies that describe the type and extent of the health surveillance programme.
   - Comparative studies that compare the effects of a health surveillance programme to no or an alternative programme including cross sectional, case-control, cohort and randomized contol trials.
2. All languages will be included;
3. All papers published after 1 January 2000 will be included;

## Search strategy

Both bibliographic databases and internet sources will be searched. Table 1 gives a summary of the information sources and Table 2 a summary of the search strategy. The references of included studies will also be scanned to identify any additional citations which were not identified through the initial search.

Table 1: Databases to be search for this systematic review

| **List of Databases** |
| --- |
| MEDLINE and/or PubMed |
| EMBASE |
| CAB extracts |
| Toxline |
| OSH Update (Reference Collection) |

**TABLE 2: SEARCH STRATEGY FOR THIS REVIEW**

| ***For health examinations***  “health examination”[tiab] OR “health examinations”[tiab] OR “health surveillance”[tiab] OR “medical surveillance”[tiab] OR biomonitoring[tiab] OR “physical examination”[tiab] OR “physical examinations”[tiab] OR checkup[tiab] OR “check up”[tiab] OR “periodic examination”[tiab] OR “periodic examinations”[tiab] OR “symptoms” [tiab] OR “signs” [tiab] OR “clinical examinations” [tiab] OR “clinical examination” [tiab] OR “special investigation” [tiab] OR “side room investigation” [tiab] OR “lung function” [tiab] OR “spirometry” [tiab] OR “chest X-ray” [tiab] OR “exhaled” [tiab]  ***For Nano***  nanomaterial*[tiab] OR “nano material”[tiab] OR “nano materials”[tiab] OR 7anoparticles* OR “nano particle” OR “nano particles” OR nanofibre*[tiab] OR “nano fibre”[tiab] OR “nano fibres”[tiab] OR nanotube*[tiab] OR “nano tube”[tiab] OR “nano tubes”[tiab] OR nanostructure*[tiab] OR “nano structure”[tiab] OR “nano structures”[tiab] OR nanofil*[tiab] OR nanowire*[tiab] OR “nano wire”[tiab] OR “nano wires”[tiab] OR nanosphere*[tiab] OR nanopowder*[tiab] OR “nano powder”[tiab] OR “nano powders”[tiab] OR nanocomposite*[tiab] OR “nano composite”[tiab] OR nanoconjugate*[tiab] OR “nano conjugate”[tiab] OR “nano conjugates”[tiab] OR enm[tiab] OR enms[tiab] OR swcnts [tiab] OR mwcnts[tiab] OR nanoiron[tiab] OR nanosilver OR nanogold OR nanoTIO2 OR titania[tiab] OR nanoSiO2 OR nanoAlO OR nanoAl2O3 OR nanoCeO OR nanoCeO2 OR fullerene* OR “carbon nanotubes”[tiab] OR dendrimer*[tiab] OR nanoclay* OR qdot*[tiab] OR “quantum dots” OR nanostructures[mh]  ***For work***  (work[tiab] OR works*[tiab] OR work’*[tiab] OR worka*[tiab] OR worke*[tiab] OR workg*[tiab] OR worki*[tiab] OR workl*[tiab] OR workp*[tiab] OR occupation*[tiab] OR job[tiab] OR employ*[tiab]) |
| --- |

## Study selection

All citations will be imported into EppiReviewer, a web-based application, where they will be reviewed and coded. Two reviewers will independently determine if the titles and abstracts that were identified in the search do not fulfill one or more of the inclusion criteria and, thus, can be excluded. The remaining articles resulting from this selection will be assessed, again, by two reviewers based on the full-text of the articles to see which articles fulfill all inclusion criteria. If there is a disagreement on inclusion it will be resolved by discussion, or, if no consensus can be reached, a third reviewer will be consulted. Duplicate studies will be removed. The resulting list will include studies from which data will be extracted.

## Data extraction and management

Two reviewers will independently extract data from the articles that are on the list of included studies. Table 3 below provides a summary of the data variables to be extracted. All data extraction will be done in duplicate by two reviewers independently. If disagreement occurs, it will be resolved by discussion, or, if no consensus can be reached, it will be resolved by involvement of a third reviewer. The studies and their characteristics will be exported and listed in an Excel table to be able to derive the most appropriate comparisons.

Table 3: Data variables to be extracted

|  | **Variable** | **Explanation/definition** |
| --- | --- | --- |
| BACKGROUND | Year of study | Date first accepted for publication |
|  | Journal of publication | Name of journal |
|  | Country of origin | country where study was conducted |
|  | conflict of interests | Did the authors state any conflict of interest |
|  | Funding | Who funded the study |
| STUDY DESIGN AND SETTING | Study Setting | 1. Industrial/commercial   e.g. laboratory  e.g. production/synthesis  e.g. application   1. Research   e.g. laboratory  e.g. production/synthesis  e.g. application |
|  | Number of employees/workers | Total number employed at company/laboratory |
|  | Description of nanomaterials | Physico-chemical properties |
|  | Study design | Observational (case study, non-systematic review/expert opinion, cross sectional, case-control, longitudinal)  Experimental (before-after; RCT)  Systematic Review/Meta-analysis |
|  | Study Participants | Number of participants;  Male: Female;  Age (mean and SE) |
|  | Possible routes of exposure | Dermal  Ingestion (GIT)  Inhalation |
|  | Hygiene measures taken | Engineering controls (brief description if available)  PPE (brief description if available) |
| INTERVENTION AND CONFOUNDERS | Intervention type: Surveillance | 1. Components – biological, clinical 2. Measures taken – e.g. urine samples; symptom questionnaires; clinical investigations CXR/LFx 3. Frequency of taking measurements 4. Method of measurement: equipment and procedure (brief description if available) 5. Healthcare worker responsible for taking measures (occupational hygienist, nurse, doctor, other) 6. Coverage: Proportion of workforce participating in surveillance programme 7. Description of action levels e.g. if symptoms present, if metals in urine etc 8. Description of how surveillance information is used 9. Any information on the cost of surveillance programme costs? |
|  | Adjusted for confounders | Smoking and/or other chemical exposures |
|  | Comparator | None, if any comparison – provide description. |
|  | Outcome | 1. Health outcomes    1. Respiratory system    2. Cardiovascular system    3. Other 2. Clinical:    1. Signs    2. Symptoms 3. Biological    1. Urine analysis    2. Blood analysis 4. Special investigations    1. Lung function    2. Imaging 5. Surveillance system    1. Coverage    2. Costs |

Where appropriate: if the outcome measure is a dichotomous outcome, then relative risks will be used as estimates of the effect of exposure. If the health effects are measured on a continuous scale Mean Differences will be used.

## Assessment of heterogeneity

First, studies will be assessed for similarity of participants, design, intervention, control and outcome measurement. NM exposure will be categorized according to following grouping: metal, metal oxides and carbon-based. We will assess outcome measurements for biological monitoring by nanomaterial group and clinical monitoring by nanomaterial group together in order to synthesis the findings (see Table 4).

Table 4: Matrix for grouping outcome measures together

| **NM group** | **Outcome measure** | |
| --- | --- | --- |
|  | **Biological** | **Clinical** |
| **Metal** | Exhaled air/urine/blood | Symptom Questionnaire/  Radiological/  lung function |
| **Metal oxides** | Exhaled air/urine/blood | Symptom Questionnaire/  Radiological/  lung function |
| **Organic** | Exhaled air/urine/blood | Symptom Questionnaire/  Radiological/  lung function |

If sufficient evidence is obtained from the review, statistical heterogeneity will be assessed by means of the I^2^ statistic. The values of I^2^ of 25%, 50% and 75% will be interpreted as low, moderate and high degrees of heterogeneity, respectively.

The studies with similar participants, interventions and outcomes will be combined in one comparison and the results pooled for statistical analyses, if possible. If this is not possible, a narrative comparison will be used to describe the studies. The outcomes will be evaluated in order to determine if variance is caused by the types of participants. In addition, it will also be determined if results differ between studies that are carried out before and after the year 2006, as well as, in participants from Western Europe and the US, versus, participants from other countries.

The results will be further evaluated in order to determine if they are sensitive to the inclusion of low quality studies, with a high risk of bias, by only including the studies with low risk of bias in the meta-analysis. Fixed-effect meta-analysis will be used to assess how sensitive the results are to the model assumptions.

## Assessment of study quality

We will assess risk of bias for each study based on the assumption that the authors had determined:

- Validity of the experimental technique used
- Sensitivity of the instrument used to generated the result
- Thoroughness of the information of the questionnaire used in the survey.

Across studies, per comparison, the GRADE approach will be used to assess the overall quality of evidence and make recommendations.[7]

All the studies will be incorporated to see any observed measurable effect. The data will be synthesized by combining studies considered to be similar by either NM group, or, the outcome measure (see Table 4). Thereafter, the studies will be compared between the NM type (metal, metal oxides or organic) and reported health problem e.g. cardiovascular, respiratory etc.

# Time lines and budget

The budget would primarily be used to obtain the EppiReviewer license (GBP 105) and the proposed time-line is indicated below.

Figure 1: Project time-lines

|  | Month | May | | | | June | | | | July | | | | August |
| --- | --- | --- | --- | --- | --- | --- | --- | --- | --- | --- | --- | --- | --- | --- |
|  | Week | 1 | 2 | 3 | 4 | 1 | 2 | 3 | 4 | 1 | 2 | 3 | 4 |  |
| Activity | Database search |  |  |  |  |  |  |  |  |  |  |  |  |  |
|  | Develop coding frame |  |  |  |  |  |  |  |  |  |  |  |  |  |
|  | Import Citations |  |  |  |  |  |  |  |  |  |  |  |  |  |
|  | Abstract and title review |  |  |  |  |  |  |  |  |  |  |  |  |  |
|  | Full text uploads |  |  |  |  |  |  |  |  |  |  |  |  |  |
|  | Full Text review |  |  |  |  |  |  |  |  |  |  |  |  |  |
|  | Data analysis |  |  |  |  |  |  |  |  |  |  |  |  |  |
|  | Report writing |  |  |  |  |  |  |  |  |  |  |  |  |  |

# References

1. WHO | Public health surveillance. In: WHO [Internet]. [cited 28 Jan 2015]. Available: http://www.who.int/topics/public_health_surveillance/en/

2. Nasterlack M. Role of medical surveillance in risk management. Journal of Occupational and Environmental Medicine. 2011;53: S18–S21.

3. Trout DB. General principles of medical surveillance: implications for workers potentially exposed to nanomaterials. Journal of Occupational and Environmental Medicine. 2011;53: S22–S24.

4. Handy RD, Shaw BJ. Toxic effects of nanoparticles and nanomaterials: Implications for public health, risk assessment and the public perception of nanotechnology. Health, Risk & Society. 2007;9: 125–144. doi:10.1080/13698570701306807

5. Kriebel D, Tickner J, Epstein P, Lemons J, Levins R, Loechler EL, et al. The precautionary principle in environmental science. Environmental health perspectives. 2001;109: 871.

6. Bergamaschi E. Human biomonitoring of engineered nanoparticles: an appraisal of critical issues and potential biomarkers. Journal of Nanomaterials. 2012;2012: 6.

7. Andrews J, Guyatt G, Oxman AD, Alderson P, Dahm P, Falck-Ytter Y, et al. GRADE guidelines: 14. Going from evidence to recommendations: the significance and presentation of recommendations. J Clin Epidemiol. 2013;66: 719–725. doi:10.1016/j.jclinepi.2012.03.013
