# Supplementary material for: Systematic Review of Screening and Surveillance Programs to Protect Workers from Nanomaterials
Source: PLoS One. 2016 Nov 9;11(11):e0166071. doi: 10.1371/journal.pone.0166071 (PMC5102462; doi:10.1371/journal.pone.0166071)
Supplement: S2 Text — (DOC) [file pone.0166071.s004.doc]

**Search Strategy:**

**For health examinations**

“health examination”[tiab] OR “health examinations”[tiab] OR “health surveillance”[tiab] OR “medical surveillance”[tiab] OR biomonitoring[tiab] OR “physical examination”[tiab] OR “physical examinations”[tiab] OR checkup[tiab] OR “check up”[tiab] OR “periodic examination”[tiab] OR “periodic examinations”[tiab]

**For Nano**

nanomaterial*[tiab] OR “nano material”[tiab] OR “nano materials”[tiab] OR nanoparticle* OR “nano particle” OR “nano particles” OR nanofibre*[tiab] OR “nano fibre”[tiab] OR “nano fibres”[tiab] OR nanotube*[tiab] OR “nano tube”[tiab] OR “nano tubes”[tiab] OR nanostructure*[tiab] OR “nano structure”[tiab] OR “nano structures”[tiab] OR nanofil*[tiab] OR nanowire*[tiab] OR “nano wire”[tiab] OR “nano wires”[tiab] OR nanosphere*[tiab] OR nanopowder*[tiab] OR “nano powder”[tiab] OR “nano powders”[tiab] OR nanocomposite*[tiab] OR “nano composite”[tiab] OR nanoconjugate*[tiab] OR “nano conjugate”[tiab] OR “nano conjugates”[tiab] OR enm[tiab] OR enms[tiab] OR swcnts [tiab] OR mwcnts[tiab] OR nanoiron[tiab] OR nanosilver OR nanogold OR nanoTIO2 OR titania[tiab] OR nanoSiO2 OR nanoAlO OR nanoAl2O3 OR nanoCeO OR nanoCeO2 OR fullerene* OR "carbon nanotubes"[tiab] OR dendrimer*[tiab] OR nanoclay* OR qdot*[tiab] OR “quantum dots” OR nanostructures[mh]

**For work**

(work[tiab] OR works*[tiab] OR work'*[tiab] OR worka*[tiab] OR worke*[tiab] OR workg*[tiab] OR worki*[tiab] OR workl*[tiab] OR workp*[tiab] OR occupation*[tiab])
